# A novel corneal epithelial cell culture assay for assessing topical antivirals against herpes simplex virus keratitis

**DOI:** 10.1016/j.bbrep.2026.102676

**Published:** 2026-06-18

**Authors:** Hidenobu Kuniyoshi, Yui Numata, Mina Sasano, Hironori Hayashi, Nobuo Fuse, Stefan G. Sarafianos, Eiichi N. Kodama

**Affiliations:** a Department of Infectious Diseases, Graduate School of Medicine, Tohoku University, 2-1 Seiryo-machi, Aoba-ku, Sendai, 980-8575, Japan; b Division of Infectious Diseases, International Research Institute of Disaster Science, Tohoku University, 2-1 Seiryo-machi, Aoba-ku, Sendai, 980-8575, Japan; c Tohoku Medical Megabank Organization, Tohoku University, 2-1 Seiryo-machi, Aoba-ku, Sendai, 980-8573, Japan; d Laboratory of Biochemical Pharmacology, Department of Pediatrics, Center for ViroScience and Cure, Emory University School of Medicine, Children's Healthcare of Atlanta, 1760 Haygood Drive NE, Atlanta, GA, 30322, USA

**Keywords:** HSV, Keratitis, Herpes, Antiviral, In vitro, Cornea, Model

## Abstract

Various in vitro models have been used to study HSV infection, but corneal epithelial-based assay systems for evaluating antiviral activity remain limited. In this study, we established a in vitro assay of HSV keratitis using the CRL-11516, an immortalized human corneal epithelial (HCE) cell line. HSV readily infected HCE cells and exerted cytopathic effects. These effects were quantified *via* formazan-based colorimetric assay, which indicated the suitability of our system for antiviral evaluation. Most clinically available antivirals exhibited sufficient activity in our model. Specifically, acyclovir and its prodrug, valaciclovir, exerted comparable effects after direct application to HCE cells. In contrast, vidarabine showed little antiviral activity. Notably, 2′-deoxycoformycin, an adenosine deaminase inhibitor, only partially restored its activity, indicating that factors other than adenosine deaminase contribute to the limited efficacy of vidarabine. These results support the widely recognized superior clinical performance of acyclovir. Our system also supported assays with thymidine kinase-deficient HSV-1, showing high application potential. Overall, the developed immortalized HCE cell-based model provides a practical platform for topical antiviral evaluation that can reduce the reliance on animal experiments for preclinical drug development.

## Highlights

• First corneal epithelial model for direct topical HSV-1/2 antiviral evaluation.
• Platform evaluates all major HSV antivirals except araA, clarifying limitations.
• Enables early ocular screening and may reduce downstream animal use.

## Glossary

ACV: acyclovir
ADA: adenosine deaminase
araA: 9-β-d-arabinofuranosyladenine
BVdU: 5-bromovinyl-2′-deoxyuridine
dCF: 2′-deoxycoformycin
GCV: ganciclovir
HCE: human corneal epithelial
HSV: herpes simplex virus
RPMI: Roswell Park Memorial Institute
TK: thymidine kinase

## 1 Introduction

Keratitis, caused by herpes simplex virus (HSV), is a serious ocular disease frequently associated with blurred vision and blindness [1]. Herpetic keratitis is predominantly caused by HSV-1; HSV-2 has also been reported to cause ocular infections, albeit less frequently [2,3]. It annually affects roughly 1.5 million people worldwide, especially in developing countries, with 40,000 new cases of severe monocular visual impairment or blindness reported every year [4]. Recent epidemiological studies have demonstrated the global burden of HSV keratitis. A systematic study estimated over 1.8 million annual cases of herpetic eye disease worldwide, including approximately one million cases of epithelial keratitis alone [5]. In developed countries, annual incidence of new or recurrent HSV keratitis is approximately 18–25 cases per 100,000 people, with the recurrence rate approaching 50% at 5 years and ≥60% at 20 years [6]. A previous longitudinal U.S. study reported an annual incidence of 11.8 new cases per 100,000 persons [7].

After diagnosis, treatment for HSV keratitis typically involves the topical and/or systemic administration of antiviral agents, such as acyclovir (ACV). When initiated promptly, these agents are highly effective in controlling viral replication, reducing inflammation, and improving the visual outcomes; they are associated with a favorable prognosis in most cases. From a patient-oriented standpoint, eye drops are generally preferred to ointments, however, ACV ophthalmic ointment typically requires application approximately five times daily [8]. Accordingly, there is a clinical need to prioritize screening antivirals suited for eye-drop formulations with prolonged ocular efficacy to reduce dosing frequency. Furthermore, prolonged or repeated use is often accompanied by disease recurrence, which remains a serious clinical challenge in the treatment of HSV keratitis. Antiviral resistance, although typically observed in immunocompromised patients, has also been reported in some immunocompetent individuals, with rates of up to 6.4% [9]. Therefore, novel antivirals are urgently needed for this disease.

Antiviral assays for HSV are traditionally performed using tumor cells and fibroblasts such as the MRC-5 cells, rather than ocular epithelial cells. Vidarabine (9-β-d-arabinofuranosyladenine [araA]), which exhibits little activity in the NC37 Burkitt's lymphoma derived B-lymphoblast cell line [10], has been successfully evaluated in the Roswell Park Memorial Institute (RPMI)-8226 and MRC-5 cells [11]. Previously, vidarabine ophthalmic ointment (3%) was widely used for acute keratitis and recurrent superficial keratitis caused by HSV-1 and HSV-2. However, subsequent clinical trials demonstrated the superior efficacy of 3% ACV ointment [12,13]. One explanation for this superior efficacy is that araA, an adenosine analog, is rapidly deaminated by cellular and extracellular adenosine deaminases (ADAs), and the deaminated product, 9-β-d-arabinofuranosyl hypoxanthine (araH), exhibits only one-tenth the activity of araA (Williams, 1977). Although ADA inhibitors, such as 2′-deoxycoformycin, partially improve araA stability in rabbit ocular tissues, their clinical relevance remains limited [14].

Previous studies have demonstrated the utility of human corneal epithelial (HCE) cell lines in investigating HSV infection [15] and antiviral responses [16]. In this study, we re-evaluated anti-HSV drugs using the CRL-11516, an immortalized human corneal epithelial (HCE) cell line. As corneal epithelial cells are the primary site of HSV infection and directly accessible for topical therapy, we established a system that acted as a better in vitro platform than conventional non-ocular cells. In addition, our system may reduce the need for subsequent animal experiments by facilitating the early screening of candidate drugs in the actual target cells.

## 2 Materials and methods

### 2.1 Cells and viruses

RPMI-8226 (human B-lymphoblastoid) cells were maintained in the RPMI-1640 medium supplemented with 10% fetal calf serum (FCS), 100 U/mL penicillin G, and 50 μg/mL streptomycin (complete RPMI medium) at 37 **°**C and 5% CO_2_ [17]. Moreover, CRL-11516 HCE cells, immortalized *via* transfection of the pRSV-T plasmid [18] containing the Rous sarcoma virus long terminal repeat and SV40 early region genes, were cultured in a collagen I-coated flask (Nunc; Thermo Fisher Scientific, Waltham, MA, USA) with the Dulbecco's modified Eagle's medium (DMEM) supplemented with 10% FCS, 100 U/mL penicillin G, and 50 μg/mL streptomycin (complete DMEM) and 50% (v/v) keratinocyte-serum free medium (Thermo Fisher Scientific). This mixed medium is hereafter referred to as the “assay medium.” Cells were maintained at 37 **°**C and 5% CO_2_ [19]. Both cell lines were purchased from the American Type Culture Collection (Manassas, VA, USA). Additionally, 3-(4,5-dimethylthiazol-2-yl)-2,5-diphenyltetrazolium bromide (MTT) was purchased from Sigma-Aldrich (St. Louis, MO, USA) and used for formazan-based colorimetric assay (MTT assay). An MTT solution (5 mg/mL in PBS) was prepared and stored at 4 **°**C.

HSV type 1 KOS, type 2 G, and thymidine kinase (TK)-deficient HSV-1 (TK^–^ HSV-1) strains were kindly provided by Shiro Shigeta (Fukushima Medical University, Fukushima, Japan). HSV stocks were prepared by inoculating RPMI-8226 cells as previously described [17]. RPMI-8226 cells (2 × 10^5^ cells/mL) were infected with each virus at an MOI of 0.1 and cultured until a pronounced cytopathic effect was observed. The culture supernatants containing viruses were harvested, clarified by centrifugation (2000 rpm, 10 min), aliquoted, and stored at −80 °C until use. Viral titers were determined in CRL-11516 cells. Serial half-logarithmic dilutions of the virus were prepared in 96-well plates (100 μL per well), after which cells (2 × 10^5^ cells/mL) were added. The plates were incubated for 5 days, and the cytopathic effects were assessed.

### 2.2 Antivirals

ACV and ganciclovir (GCV) were purchased from Wako (Shiga, Japan), valacyclovir (Val-ACV) and valganciclovir (Val-GCV) from Tokyo Chemical Industry (Tokyo, Japan), and araA, 5-bromovinyl-2′-deoxyuridine (BVdU), cidofovir ((S)-1-(3-hydroxy-2-phosphonylmethoxypropyl) cytosine; HPMPC or CDV), dextran sulfate (MW 5000; DS5000), and phosphonoformic acid (foscarnet) from Sigma-Aldrich. Additionally, ADA inhibitor 2′-deoxycoformycin (dCF; pentostatin), a tight-binding transition-state analog potently inhibiting ADA at nanomolar concentrations, was obtained from Yamasa Corporation (Choshi, Japan). All compounds were dissolved in DMSO, and its final concentration in the assay was maintained below 0.5%. This had no significant effect on cell viability.

### 2.3 Anti-HSV assay

Inhibitory effects of the laboratory and clinically approved compounds on HSV replication were examined by assessing the inhibition of HSV-induced cytopathic effects on CRL-11516 cells. Briefly, CRL-11516 cells were treated with trypsin (Sigma-Aldrich, MO, USA), suspended at 2x10^5^ cells/mL, and exposed to viruses at a multiplicity of infection (MOI) of 0.01. Immediately after viral exposure, the cell suspension (2 × 10^4^ cells/mL in 100 μL) was placed in each well of a collagen type I-coated 96-well flat microtiter culture plate (IWAKI, AGC Techno Glass Co., Ltd, Shizuoka, Japan) containing various concentrations of test compounds (100 μL), resulting in a final assay volume of 200 μL and the indicated final concentrations. After incubation for five days, the number of viable cells was determined *via* MTT assay, a formazan-based colorimetric assay, as previously described [[20], [21], [22]]. An MTT solution (5 mg/mL in PBS) was added to the culture medium at one-tenth of the total volume to yield a final concentration of 0.5 mg/mL. Compound cytotoxicity was also assessed *via* MTT assay. After incubation with the MTT solution, 120 μL of the reaction mixture (total volume 220 μL) was carefully aspirated without disturbing the cell monolayer. Subsequently, 100 μL of MTT solubilization solution was added to each well to dissolve the formazan crystals. All experiments were performed in triplicate. The OD values of the MTT assay were measured using a BioTek 800 TS microplate reader (Winooski, VT, USA). The EC_50_ value was the concentration of the compound required to achieve 50% protection against virus-induced cytopathic effects relative to the infected untreated controls. EC_50_ values were determined from dose–response data and are summarized as the mean ± SD for at least three independent experiments.

**Fig. 1:**
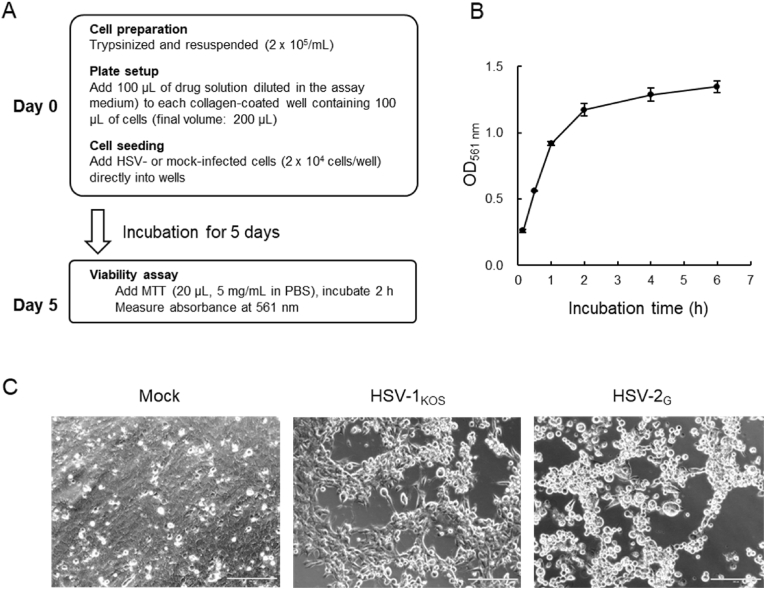
Workflow of the anti-herpes simplex virus (HSV) assay, 3-(4,5-dimethyl-2-thiazolyl)-2,5-diphenyltetrazolium bromide (MTT) conversion of mock-infected CRL-11616 cells, and microscopic profile of virus-infected CRl-11516 cells. (A) Simplified timeline of the experimental workflow. HSV- and mock-infected cells (2 × 10^5^ cells/mL; 100 μL/well) were directly seeded in collagen-coated 96-well plates containing pre-diluted compounds, without prior cell attachment, to streamline the assay workflow. After five days of incubation, cell viability was assessed using the MTT assay (5 mg/mL in phosphate-buffered saline [PBS]) for 2 h, and the reaction was stopped at the indicated time points using the MTT reaction stop solution (isopropanol with 2% Triton-X100 and 0.2 N HCl). Then, MTT conversion profile was measured using a microplate reader at a wavelength of 561 nm. (B) CRL-11516 cells efficiently converted MTT into formazan. OD_561_ values reached over 1.0 after 1.5 h of incubation with MTT. (C) Cells were infected at an MOI of 0.01 and incubated for XX days. Representative images showing typical cytopathic effects under these conditions are presented. Microscopic observations of CRL-11516 cells incubated with the mock and HSV-1 and -2 (MOI of 0.01) for four days. Representative images showing typical cytopathic effects under these conditions are presented. Scale bars, 200 μm.

The entire workflow of our established procedure is summarized in Fig. 1A, which illustrates the sequence of major steps in a simplified form.

### 2.4 MTT assay and virus titration assay

CRL-11516 cells were seeded in 96-well plates and infected with HSV at an MOI of 0.01 in the presence of acyclovir (ACV) at the indicated concentrations (0.1–100 μM). Mock-infected controls were also included. Each condition was tested in triplicate.

After 5 days of incubation, 50 μL of culture supernatants were collected from each well and subjected to viral titration. Serial half-logarithmic dilutions of the supernatants were prepared, starting from a 1:100 dilution, and inoculated into CRL-11516 cells in 96-well plates. Following 5 days of incubation, cytopathic effects were evaluated, and viral titers were determined using the TCID_50_ method. Since the collected culture supernatants were initially diluted to 1:100 before titration and subjected to further serial dilutions, the residual ACV concentration during the infectivity assay was considered negligible. Therefore, carryover effects of ACV on the subsequent TCID_50_ assay were unlikely to influence the measured viral titers.

### 2.5 Inhibition of ADA activity

Next, araA was diluted in the presence of dCF at concentrations of 0.2, 2, and 20 μM, which were reduced to final concentrations of 0.1, 1, and 10 μM, respectively, after mixing with the cells. We previously showed that dCF is sufficient to inhibit ADA at 2.5 μM in cell-based experiments [23]. Here, serial dilutions were prepared, and similar to that in the MTT assay, either mock- or HSV-infected cells were seeded in the collagen I-coated 96-well plate. The cultures were maintained for five days to evaluate whether dCF enhanced the anti-HSV activity in CRL-11516 cells. Wells containing dCF without araA were also examined to determine the impact of dCF alone.

## 3 Results

### 3.1 Formazan formation in CRL-11516 cells

To examine the potential conversion of MTT into formazan in CRL-11516 cells, MTT reagent (5 mg/mL) was added to each well of the CRL-11516 culture plate on day 5. Indeed, MTT was successfully converted into formazan (Fig. 1). Absorbance at 561 nm markedly increased during the initial 2-h incubation, showing only a slight increase thereafter (Fig. 1B). These results suggest that a 2-h incubation period is sufficient for accurate cell viability assessment.

### 3.2 Cytopathic effects of HSV-1 and HSV-2 on CRL-11516 cells

In mock-infected cultures, CRL-11516 cells exhibited the typical morphology of HCE cells, forming a uniform spindle-shaped monolayer [18,19]. In contrast, cells infected with HSV-1 or HSV-2 exhibited prominent cytopathic features, characterized by cell rounding, swelling, and detachment from the monolayer (Fig. 1C). Notably, comparable morphological alterations were observed in cells infected with HSV-1 and HSV-2. Although distinct giant cell formation was infrequent, occasional syncytium-like structures were observed. These results suggest that the susceptibility of CRL-11516 cells to HSV-1 and HSV-2 infections is sufficient for antiviral assays.

### 3.3 Anti-herpes compound activity

**Fig. 2:**
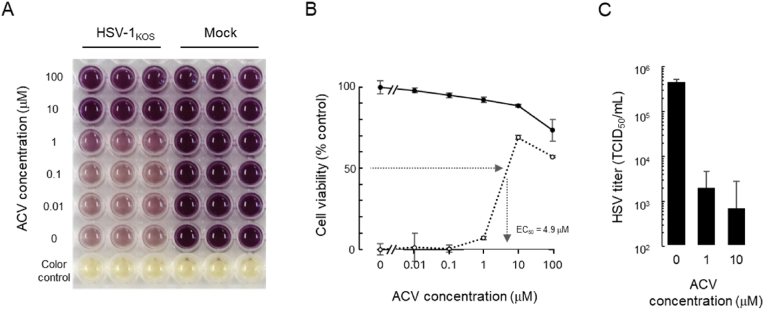
**Dose–response analysis of acyclovir (ACV) in the corneal epithelial cell assay.** (A) Representative plate images after HSV-1 infection and treatment with serial concentrations of ACV. (B) CRL-11516 cells were infected with HSV at an MOI of 0.01 in the presence of the indicated concentrations of ACV. After 5 days of incubation, cell viability was assessed using the MTT assay. Data are summarized as mean ± SD (n = 3). EC_50_ was determined using nonlinear regression analysis. Concentration–response curves show cell viability (%) in infected (dashed line) and mock-infected (solid line) cells. (C) Reduction in HSV titer following ACV treatment. Supernatants were diluted at 1:100 before titration, minimizing potential ACV carryover effects.

Antiviral activities of the representative compounds against HSV-1 and HSV-2 were evaluated in CRL-11516 cells, and the results are summarized in Table 1. To further illustrate the assay performance and derivation of EC_50_ values, a representative dose–response analysis of ACV is shown in Fig. 2. Representative plate images demonstrated concentration-dependent protection against HSV-1-induced cytopathic effects (Fig. 2A), and nonlinear regression analysis of the corresponding concentration–response data enabled calculation of the EC_50_ value (Fig. 2B). In addition, ACV treatment reduced infectious virus titers in a concentration-dependent manner, further confirming the antiviral activity detected in this assay system (Fig. 2C).

**Table 1:** Anti-herpes simplex virus (HSV)-1 and HSV-2 activities of the representative compounds in CRL-11516 cells.

Compounds	EC_50_ (μM)		CC_50_ (μM)
	HSV-1	HSV-2	
DS5000	4.6 ± 1.8	1.3 ± 0.01	>100
araA	>100	>100	>100
ACV	5.0 ± 1.8	4.4 ± 2.8	>100
Val-ACV	3.6 ± 0.4	2.7 ± 0.3	>100
GCV	0.42 ± 0.039	0.25 ± 0.02	72 ± 13
Val-GCV	0.40 ± 0.01	0.54 ± 0.13	73 ± 30
BVdU	0.50 ± 0.17	52.2 ± 16.1	>100
CDV	7.9 ± 1.9	3.5 ± 0.4	45 ± 10
PFA	186 ± 45	97 ± 34	446 ± 193

EC_50_, 50% effective concentration required to inhibit virus-induced cytopathic effects; CC_50_, 50% cytotoxic concentration. Data are represented as the mean ± standard deviation (SD) of three independent experiments. Values > 100 indicate that the tested concentrations did not reach the 50% endpoint. Abbreviations: DS5000, dextran sulfate MW 5000; araA, 9-β-d-arabinofuranosyladenine or vidarabine; ACV, acyclovir; Val-ACV, valacyclovir; GCV, ganciclovir; Val-GCV, valganciclovir; BVdU, 5-bromovinyl-2′-deoxyuridine; CDV, cidofovir; PFA, phosphonoformic acid or foscarnet.

Among the tested compounds, GCV and Val-GCV showed the most potent antiviral activities, with 50% effective concentration (EC_50_) values of 0.42 and 0.4 μM, respectively, against HSV-1 and 0.25 and 0.54 μM, respectively, against HSV-2. Both compounds exhibited relatively low cytotoxicity, with 50% cytotoxic concentrations >70 μM. BVdU exhibited strong activity against HSV-1 (EC_50_ = 0.5 μM) but was markedly less effective against HSV-2 (EC_50_ = 52 μM), consistent with previous reports that BVdU exhibits weak activity against HSV-2 [24]. In contrast, CDV and foscarnet (phosphonoformic acid; PFA) exhibited only moderate to weak activity, particularly against HSV-1. ACV and Val-ACV showed consistent activity against both HSV-1 and HSV-2. Entry inhibitor DS5000 also showed comparable activity to ACV. Notably, araA exhibited no appreciable activity, with EC_50_ values > 100 μM. All tested compounds, except CDV and GCV/ValGCV, showed 50% cytotoxic concentrations >100 μM, indicating their low cytotoxicity in CRL-11516 cells.

Taken together, these results indicate that antiviral activity can be reliably assessed using CRL-11516 cells, demonstrating that our assay system is comparable to conventional methods.

### 3.4 Effect of ADA inhibition on araA antiviral activity

**Fig. 3:**
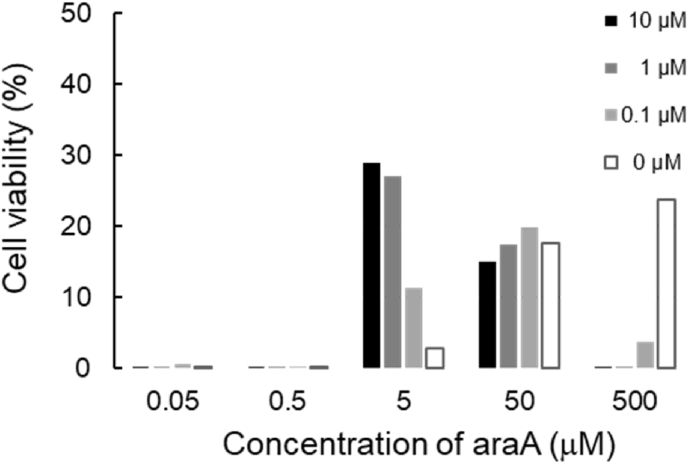
Effect of 2′-deoxycoformycin (dCF) on the anti-HSV-1 activity of 9-β-d-arabinofuranosyladenine (araA). CRL-11516 cells (2 × 10^5^ cells/mL; 100 μL/well) were infected with HSV-1 (MOI = 0.01) and treated with araA (100 μL) at 0.05–500 μM concentrations, alone or in combination with 10, 1, 0.1, and 0 μM of dCF (black, dark gray, light gray, and white bars, respectively). This resulted in a final assay volume of 200 μL. Cell viability was assessed 5 days post-infection using the MTT assay. AraA alone exhibited moderate dose-dependent antiviral activity, as reflected by the increased cell survival. Co-treatment with dCF partially restored the efficacy of araA, suggesting that adenosine deaminase (ADA)-mediated deamination contributes to the loss of antiviral activity. Although dCF enhanced the antiviral activity of araA, it simultaneously increased the cytotoxicity; however, the overall selectivity index remained unchanged.

Recent data suggest that araA is rapidly deaminated to araH by ADA in the serum, with an estimated half-life of approximately 60 min and markedly reduced antiviral activity [25]. In this study, AraA alone exhibited only moderate dose-dependent antiviral activity against HSV-1 (Fig. 3). Its activity was only marginally enhanced *via* co-treatment with ADA inhibitor dCF, suggesting that ADA-mediated deamination does not significantly limit its efficacy in this system. These findings partially support previous classic ocular studies on rabbits showing that co-administration of dCF increases the ocular araA exposure and slows its deamination, thereby enhancing its apparent antiviral activity [14]. Although these effects support the pharmacological plausibility of ADA inhibition, clinical comparisons have consistently favored ACV over araA [26], suggesting that ADA-mediated deamination is only one of several factors affecting araA efficacy in the eyes.

### 3.5 Drug susceptibility of TK-deficient (TK^−^) HSV

**Fig. 4:**
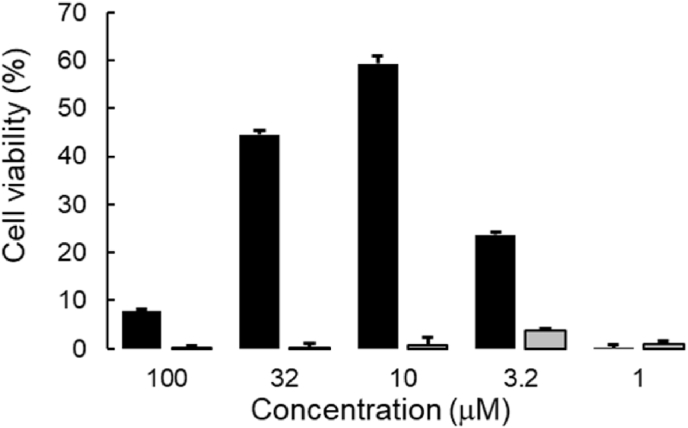
Differential effects of acyclovir (ACV) and cidofovir (CDV) on thymidine kinase-deficient (TK^−^) HSV-infected cells. CRL-11516 cells were infected with TK^−^ HSV-1 cells and treated with serial 0.5-log dilutions of CDV (black bars) or ACV (gray bars) at the indicated concentrations. Cell viability was measured *via* MTT assay 120 h after infection. Data are represented as the mean ± standard deviation (SD) of triplicate experiments. CDV, which does not require viral TK for activation, retained its antiviral activity, whereas ACV exerted no protective effects on TK^−^ HSV-infected cells.

For further application of our assay system to drug-resistant viruses, such as TK^−^ HSV-1, we evaluated the antiviral activities of CDV and ACV. As shown in Fig. 4, CDV, which does not require viral TK for activation, exhibited comparable antiviral activity in TK^−^ HSV-1-infected cells, whereas ACV showed no protective effects. Therefore, our assay reliably distinguished between TK-dependent and -independent antivirals, showing extended utility beyond wild-type HSV strains.

## 4 Discussion

In line with previous studies using HCE cell lines [16], our results support the utility of corneal epithelium-derived cells for antiviral evaluation and suggest that CRL-11516 cells provide a practical system for assessing antiviral activity against HSV-1 and HSV-2. Their susceptibility to infection and reproducible cytopathic effects, together with the expected antiviral profiles of well-characterized entry and DNA polymerase inhibitors, support the reliability of our assay platform. In addition, the representative ACV dose–response experiment confirmed that the assay can capture both cytoprotective effects and suppression of viral production. Among the compounds tested, araA exhibited weak antiviral activity, with the addition of potent ADA inhibitor dCF resulting in marginal improvement. These results suggest that factors other than ADA-mediated deamination contribute to the limited efficacy of araA, without compromising the overall robustness of our assay system.

Classic ocular studies have shown that dCF stabilizes araA in ocular tissues and enhances its apparent antiviral activity [14]. In our HCE model, dCF only partially rescued araA activity, suggesting that ADA-mediated deamination is not the sole limitation of this compound. Additional factors, such as cellular uptake, phosphorylation-dependent activation, and the intrinsically lower potency of araA compared to that of ACV in vitro [27], possibly also contributed to the observed effects. Previous studies suggest that the development of improved araA analogs or advanced drug delivery systems [25,28] can overcome the proposed barriers and renew the interest in classic antiviral scaffolds.

Our assay system is applicable to TK^−^ HSV-1 assay evaluation and has important clinical implications. ACV resistance caused by TK deficiency poses a serious problem, particularly in immunocompromised patients receiving prolonged antiviral therapy. The prevalence of ACV-resistant HSV infections reaches up to 17% in allogeneic hematopoietic stem cell transplant recipients [29]. Similarly, in HIV-positive individuals and solid organ transplant recipients, resistance rates range from 3.5 to 10% [30]. Our results suggest that CDV, but not ACV, retains its activity, highlighting the value of this platform for evaluating drug-resistant HSV strains. Therefore, CRL-11516 cell-based assay is not only effective for assessing standard antivirals but also versatile enough to support drug discovery and preclinical drug testing targeting specific clinically significant resistant variants.

Several in vitro models have been used to study HSV infection in ocular tissues, including immortalized human corneal epithelial cells (HCECs) and ARPE-19 cells [16]. These models provide valuable systems for investigating viral infections in corneal and retinal contexts. Compared with these established models, CRL-11516 cells offer a practical and reproducible platform for evaluating antiviral activity in corneal epithelium-derived cells. However, differences in the cellular properties of these models may influence viral replication and drug responses, and further comparative studies are required to fully evaluate their relative advantages.

Prodrugs such as Val-ACV require intracellular conversion by cellular enzymes to ACV, which is further phosphorylated to the active triphosphate form. The efficiency of this process varies for cell types depending on the expression of esterases and related hydrolases including valacyclovir hydrolase (BPHL) [31], as well as cellular uptake mechanisms [32,33]. In the present study, CRL-11516 cells appeared to efficiently convert Val-ACV to ACV, resulting in antiviral activity comparable to that of ACV (Table 1). Similarly, Val-GCV demonstrated antiviral activity comparable to that of GCV. This observation is consistent with the pharmacological properties of these agents as oral prodrugs, for which the valine ester moiety enhances gastrointestinal absorption *via* peptide transporters such as hPEPT1 [32,33], while intracellular esterases mediate conversion to active nucleoside analogs. Accordingly, the intrinsic antiviral potency of the prodrugs is expected to be comparable to that of their parent compounds in vitro.

In contrast, previous studies have reported limited Val-ACV activity in some cell lines such as Vero cells, which may reflect reduced uptake and/or prodrug activation capacity. Consistent with this, efficient activation of Val-ACV has been reported in epithelial-derived cells such as intestinal Caco-2 cells [33]. Furthermore, the comparable in vitro antiviral activities of acyclovir and valacyclovir against HSV-1 and HSV-2 have been demonstrated in other human cell systems under standardized conditions [34]. Taken together, these findings suggest that the antiviral activity of Val-ACV is influenced by cell-type-specific factors involved in prodrug activation and support the use of CRL-11516 cells as a practical system for evaluating antiviral activity.

Use of HCE cells provided a distinct advantage as the antiviral effects observed in vitro were more likely to accurately reflect the ocular surface environment, particularly under conditions relevant to topical administration. Unlike non-ocular cells, HCE cells enabled the evaluation of antiviral activity without the confounding influences of systemic absorption, metabolism, and tissue distribution. This feature enhances the translational relevance of our assay, facilitating more accurate approximation of the clinical scenario in which antiviral eye drops are directly applied to the corneal surface. Moreover, our model directly addresses a critical limitation in current preclinical ocular antiviral evaluation. Primary corneal cell or explant-based assays require obtaining fresh corneal tissue from animals for each preparation, which not only constitutes animal experimentation but also introduces substantial variability due to donor- and preparation-dependent differences. In contrast, the immortalized CRL-11516 HCE model eliminates the need for animal-derived corneal tissue, providing a stable, reproducible, and ethically favorable platform that fully aligns with the 3Rs principles (replacement, reduction, and refinement) [35]. Accordingly, this model represents a replacement for animal-dependent ocular infection assays, rather than a mere reduction in animal use. It provides a practical and versatile platform for anti-HSV activity assessment, enabling the identification of new antiviral candidates, refinement of therapeutic strategies, and advancement of preclinical development while promoting a more ethical and standardized approach to antiviral research.

## Funding

This work was supported by JSPS KAKENHI [JP23H05465, JP23K06901 to E.N.K, and JP25K01957] and the AMED-CREST program [grant number JP25gm1610007] of the Japan Agency for Medical Research and Development (AMED).

## CRediT authorship contribution statement

**Hidenobu Kuniyoshi:** Formal analysis, Investigation, Methodology, Writing – original draft. **Yui Numata:** Investigation, Methodology. **Mina Sasano:** Investigation, Methodology. **Hironori Hayashi:** Writing – original draft, Writing – review & editing. **Nobuo Fuse:** Writing – original draft, Writing – review & editing. **Stefan G. Sarafianos:** Writing – original draft, Writing – review & editing. **Eiichi N. Kodama:** Conceptualization, Funding acquisition, Investigation, Methodology, Resources, Supervision, Writing – review & editing.

## Declaration of competing interest

The authors declare that they have no known competing financial interests or personal relationships that could have appeared to influence the work reported in this paper.

## Data Availability

Data will be made available on request.
